# Improving postoperative outcomes in gynecologic oncology through a standardized telehealth intervention: experience from an urban safety-net hospital system

**DOI:** 10.1016/j.gore.2026.102138

**Published:** 2026-06-09

**Authors:** Lilla Markel, Lauren Ellis, Aubrey Wetzelberger, Matthew L. Anderson, Thomas Rutherford, Adrian Kohut, Maya Yasukawa, Emily Coughlin, Rahul Mhaskar, Vaagn Andikyan

**Affiliations:** a Department of Obstetrics and Gynecology, University of South Florida, Tampa, FL, United States of America; b University of South Florida Morsani College of Medicine, United States of America; c Tampa General Hospital Cancer Institute, Tampa, FL, United States of America; d Division of Gynecologic Oncology, Department of Obstetrics and Gynecology, University of South Florida Morsani College of Medicine, Tampa, FL, United States of America

**Keywords:** Postoperative care, Phone calls

## Abstract

**Objective:**

To evaluate the impact of a standardized postoperative phone call program on outcomes among patients undergoing gynecologic oncology surgery at an urban safety-net tertiary care center.

**Methods:**

As a quality improvement initiative, routine postoperative calls were implemented in January 2025 for all surgical patients on the gynecologic oncology service at our center. Nursing staff contacted patients 48–72 h after discharge using a structured questionnaire. We conducted a retrospective cohort study of patients who underwent surgery in the 4 months before and after implementation (9/1/2024–4/30/2025). Data were collected via chart review. Primary outcomes were readmission and mortality within 90 days. Secondary outcomes included ED visits, postoperative visit attendance, and outpatient complication management.

**Results:**

Among 466 patients, 215 received phone calls and 251 did not. Baseline characteristics were similar. Patients receiving calls had higher postoperative visit attendance (94.4% vs. 86.9%, *p* = 0.006) and more outpatient management of complications (18.1% vs. 6.7%, *p* < 0.001). ED visits and readmission rates were similar between groups; however, repeat readmissions were lower among contacted patients (mean 1.9 vs. 2.4, *p* = 0.042). Of those contacted, 41.4% reported concerns, most commonly nonspecific symptoms, pain, and constipation. Mortality was 1.4% in the phone call group vs. 4.4% among those who were not called (*p* = 0.059).

**Conclusions:**

Standardized postoperative phone calls improved follow-up adherence and outpatient complication management, suggesting enhanced care coordination.

## Highlights

• Standardized phone calls improved postoperative visit attendance.
• Calls increased outpatient management of complications and decreased repeat readmissions.
• 41% of patients reported concerns during follow up calls.
• Low cost, scalable phone calls can improve postoperative care coordination.

## 1 Introduction

Gynecologic oncology patients often experience complex medical and psychosocial challenges, making postoperative contact with patients critical in recognizing complications, clarifying ongoing questions regarding the recovery period, and improving the patient's experience with the care team (Salley et al., 2024; Boitano et al., 2023). These efforts can be hindered through limited access to care, ineffective communication, and prolonged time to follow-up. At our institution, which is a large tertiary care center and safety net hospital, standardized phone calls were implemented as an effort to enhance communication in the postoperative period.

Structured postoperative phone calls have been demonstrated to be a cost effective and feasible measure to maintain continuity of care, identify risk factors, improve health equity, and potentially reduce postoperative readmission across numerous surgical specialties (Salley et al., 2024; Boitano et al., 2023; Hornick et al., 2016; Goehner et al., 2021; Liu et al., 2021). Furthermore, empowering patients to actively participate in their care through open communication with their clinical care team can lead to decreased anxiety about the healing process, streamline steps toward recovery, and lead to increased patient satisfaction (Boitano et al., 2023; Goehner et al., 2021; Kenawy et al., 2023). This intervention allows for an established line of communication for early evaluation of recovery and clarification of discharge instructions. Although postoperative phone calls offer an opportunity to identify patient concerns and early adverse outcomes and guide timely intervention, their specific utility within the gynecologic oncology population remains poorly defined (Boitano et al., 2023).

We aimed to evaluate the effect of a standardized postoperative phone call program on postoperative outcomes of patients undergoing surgery on a gynecologic oncology service at an urban safety net tertiary care center.

## 2 Methods

We conducted a retrospective cohort study of patients who underwent surgery with the gynecologic oncology department at a tertiary care center between 9/1/2024 to 4/30/2025 to determine the impact of a standardized postoperative phone call initiative. The study was approved by the institutional review board.

### 2.1 Phone calls

The standardized postoperative phone call program was implemented as a quality improvement initiative for all providers beginning January 2025. All patients who underwent surgery with a gynecologic oncologist were called by nursing staff 48–72 h following discharge with predetermined questions regarding pain, bowel function, fever, wound status, bleeding, medications, and upcoming appointments (Appendix A). This was also an opportunity to address any ongoing concerns or questions, which were appropriately escalated to the clinical team of physicians and advanced practice providers for management.

### 2.2 Population

The study population included patients aged 18 years or older who underwent surgery with the gynecologic oncology team during the study window, which included the four months preceding the implementation of standardized calls and the initial four months after implementation.

### 2.3 Data collection

Data were obtained through chart review of the electronic medical record. Demographic variables included age, race, ethnicity, body mass index, insurance status, and medical comorbidities. Diagnosis categories were benign (including dysplasia), uterine cancer, ovarian cancer, cervical cancer, vulvar cancer, vaginal cancer, and non-gynecologic cancer. Patients were classified by disease site and all histological subtypes were included. Type of surgery was also obtained and categorized into abdominal hysterectomy, minimally invasive hysterectomy (including laparoscopic and robotic surgery), vaginal hysterectomy, tumor debulking surgery, hyperthermic intraperitoneal chemotherapy (HIPEC), other open procedure, other minimally invasive procedure, vulvar procedure, minor procedures (including exam under anesthesia, biopsies, D&C, hysteroscopy, cold knife cone, laser ablation, etc.). For patients who underwent multiple procedures during the study time frame, either the most major procedure or initial operation was included. For patients who received calls, phone call information was recorded, including responses to the standardized questionnaire and additional patient concerns. Outcomes included postoperative clinic follow-up, emergency department visits within 90 days, hospital readmission within 90 days, and 90-day mortality. All data was collected in Redcap.

### 2.4 Statistical analysis

Patients were divided based on whether they received a standardized phone call. The cohort that did not receive a call consisted predominantly of patients in the pre-implementation period, with a small subset of post-implementation patients who could not be reached despite attempted outreach. The groups were compared using Pearson's chi-square test for categorical variables and Fisher's exact test in cases of low cell counts. Continuous variables including age, BMI, postoperative day of discharge, and duration and number of readmissions were assessed using Mann-Whitney *U* tests. A significance level of 0.05 was used for all tests. Analysis was conducted in SPSS Version 30.

## 3 Results

A total of 466 patients underwent surgery with the gynecologic oncology department during the designated timeframe. 228 underwent surgery in the post-intervention period, of which 215 (94%) were successfully contacted. Of the 251 patients that did not receive calls, 238 were in the pre-intervention cohort and 13 were unable to be reached. There was no difference between the groups with regards to race, ethnicity, age, BMI, or diagnosis prompting surgery (Table 1). Patients in the phone call group were more likely to have private insurance than those who did not receive calls (61.7% vs. 52.4%, *p* = 0.041) (Table 1). There was no difference in the presence or type of comorbidities (Table 1). There was also no difference between the groups in the rate of surgeries classified as “Major” (Table 2). The phone call group had more minimally invasive hysterectomies and fewer vaginal hysterectomies and minor procedures (Table 2). There was no difference in abdominal hysterectomies, tumor debulking surgeries, HIPEC, other open procedure, other minimally invasive procedures, or vulvar procedures (Table 2).

**Table 1:** Baseline patient characteristics.

		Phone Call		*p*-value
		No (*N* = 251)	Yes (*N* = 215)	
Race				0.929
	White	162 (65.3)	140 (66.0)	
	Black	43 (17.3)	34 (16.0)	
	Asian	0 (0.0)	0 (0.0)	
	AIAN	0 (0.0)	0 (0.0)	
	Other	43 (17.3)	38 (17.9)	
Hispanic				0.100
	No	182 (73.1)	140 (66.0)	
	Yes	67 (26.9)	72 (34.0)	
Insurance				0.041*
	Private	130 (52.4)	129 (61.7)	
	Medicare	59 (23.8)	48 (23.0)	
	Medicaid	32 (12.9)	15 (7.2)	
	None	4 (1.6)	7 (3.3)	
	Other	23 (9.3)	10 (4.8)	
Age *(mean ± SD)*		53.0 ± 14.5	53.8 ± 14.5	0.651
BMI *(mean ± SD)*		31.3 ± 9.5	30.5 ± 8.5	0.736
Diagnosis				
	Benign	137 (54.6)	131 (60.9)	0.167
	Uterine cancer	49 (19.5)	39 (18.1)	0.708
	Ovarian cancer	34 (13.5)	25 (11.6)	0.532
	Cervical cancer	25 (10.0)	13 (6.0)	0.124
	Vulvar cancer	5 (2.0)	6 (2.8)	0.572
	Vaginal cancer	2 (0.8)	1 (0.5)	1.000
	non-GYN cancer	3 (1.2)	4 (1.9)	0.709

**Table 2:** Type of surgery.

Types of surgery		Phone Call		p-value
		No (N = 251)	Yes (N = 215)	
Surgery Classification				0.148
	Minor†	77 (30.7)	53 (24.7)	
	Major	174 (69.3)	162 (75.3)	
Open abdominal hysterectomy		76 (30.3)	55 (25.6)	0.262
Minimally invasive hysterectomy		48 (19.1)	64 (29.8)	0.007*
Vaginal hysterectomy		8 (3.2)	1 (0.5)	0.042*
Tumor debulking surgery		11 (4.4)	16 (7.4)	0.158
HIPEC		17 (6.8)	7 (3.3)	0.087
Other open procedure		14 (5.6)	19 (8.8)	0.171
Other minimally invasive procedure		8 (3.2)	13 (6.0)	0.138
Vulvar procedure‡		11 (4.4)	10 (4.7)	0.887
Minor procedure§		58 (23.1)	30 (14.0)	0.012*

† Includes minor laparoscopic procedures (ex. BSO), vulvar procedures, other minors as described below.

‡ Includes vulvectomy, wide local excision.

§ Includes exam under anesthesia, biopsies, D&C, hysteroscopy, cold knife cone, laser ablation.

Of those patients receiving standardized calls, 41.4% reported a concern. The most common reason was “other” (22.3%), which included concerns such as nausea/vomiting, bloating, questions regarding foley, FMLA paperwork, etc. The next most frequent concerns were pain (13.5%) and constipation (7.4%). Fever, bleeding, wound, prescription, or appointment related concerns were reported by less than 5% of patients (Table 3).

**Table 3:** Phone call concerns.

Concerns during phone call	N	%
Pain	29	13.5%
Constipation	16	7.4%
Appointment	9	4.2%
Prescription	6	2.8%
Wound concern	3	1.4%
Bleeding	2	0.9%
Fever	1	0.5%
Other	48	22.3%
None	126	58.6%

Patients who received a phone call were more likely to attend a postoperative visit (94.4% vs. 86.9%, p = 0.006). There was no difference in ED visits (27% in phone call group vs. 26.3%, *p* = 0.868; Table 4) or reasons for ED visit. There was also no difference in readmission rates (17.7% in phone call group vs. 19.9%, *p* = 0.537), however, among those readmitted multiple times, patients who received a phone call had a fewer number of readmissions (1.9 +/− 1.5 vs. 2.4 +/− 1.5, *p* = 0.042). There was no difference in reasons for readmissions. Patients who received a phone call were more likely to have complications such as pain or wound concerns addressed outpatient (18.1% vs. 6.7%, *p* < 0.0001; Table 4).

**Table 4:** Outcomes.

	Phone Call		p-value
	No (*N* = 251)	Yes (N = 215)	
Patient initiated phone call *(n(%))*	121 (48.2%)	96 (44.7%)	0.443
Attended postop visit *(n(%))*	218 (86.9%)	203 (94.4%)	0.006*
ED visit *(n(%))*	66 (26.3%)	58 (27.0%)	0.868
Hospital Readmission *(n(%))*	50 (19.9%)	38 (17.7%)	0.537
Duration of readmission (*mean ± SD)*	7.6 ± 6.9	5.6 ± 4.6	
Multiple readmissions *(n(%))*	18 (36.7%)	12 (31.6%)	0.616
Number of readmissions (*mean ± SD)*	2.4 ± 1.5	1.9 ± 1.5	0.042*
Complications addressed outpatient *(n(%))*	17 (6.7%)	39 (18.1%)	<0.001*
Death *(n(%))*	11 (4.4%)	3 (1.4%)	0.059

In the 90 days after surgery, there were 11 deaths (4.4%) among those who were not called and 3 (1.4%) among those who received a phone call (p = 0.059). Causes of death in the phone call cohort included progression of disease/hospice (*n* = 5), bowel perforation (*n* = 2), aspiration (*n* = 1), septic shock (n = 1), tumor lysis syndrome (n = 1), and unknown (n = 1). In the phone call group, 1 patient died in hospice, and 2 patients died of aspiration pneumonia.

## 4 Discussion

In this study, implementation of a standardized postoperative phone call in a gynecologic oncology population was associated with improvements in postoperative care engagement and complication management. Given the complexity and potential risks associated with these surgeries, timely communication helps detect issues that may not prompt immediate patient-initiated contact.

One of the most notable findings was improved postoperative visit attendance among patients who received standardized calls. Among those who received a phone call, 94.4% attended a postoperative visit, compared to 86.9% of those who were not called (*p* = 0.006). As a tertiary care center, many patients enter care through the ED or urgent transfers, often bypassing standard clinic pathways and leading to less structured postoperative follow up. Implementing postoperative phone calls can help bridge this gap and ensure more consistent continuity of care. For oncology patients in particular, surveillance and timely initiation of adjuvant therapy are critical to long term patient outcomes.

While there was no difference in overall ED visits or readmission rates, patients receiving postoperative calls had fewer repeat readmissions, suggesting that early intervention may help prevent recurrent issues requiring hospitalization. A study in vascular surgery patients similarly found no difference in readmission rates with postoperative calls, however, they were able to identify risk factors for readmission, such as continued prescription pain medication use and continued dressing changes (Hornick et al., 2016). Other studies have demonstrated reduction in readmissions with postoperative outreach. In a prospective study looking at vascular surgery patients, those who received a postoperative phone call had lower readmission rates compared to those who were not contacted (Salley et al., 2024). A study examining mobile health patient engagement technology (PET) in gynecologic oncology patients similarly found lower readmission rates, although the intervention was delivered through a digital application rather than telephone-based outreach (Boitano et al., 2023). In the benign gynecology population, a retrospective analysis found no difference in ED visits with postoperative phone calls from the surgical team, however, there was a lower rate of inpatient admissions in the phone call group (Liu et al., 2021). Importantly, while readmission rates are often viewed as an important metric, no single intervention has been proven to reduce readmission rates (Salley et al., 2024; Hansen et al., 2011), thus, postoperative phone calls should be utilized in conjunction with other methods as part of a broader multifaceted strategy.

Individuals in the phone call group had a higher likelihood of having pain or wound concerns successfully addressed in the outpatient setting (18.1% vs. 6.7%, *p* < 0.0001). This reflects improved utilization as concerns are recognized, triaged, and resolved promptly in the outpatient setting, preventing unnecessary emergency evaluations. In a study of vascular surgery patients, one fifth of the patients who were called identified a concern, but >90% could be addressed over the phone (Salley et al., 2024). There was no difference in patient initiated calls in our population (Table 4), however, a study in the urogynecology population found a reduction in patient-initiated calls (Iwanoff et al., 2019). This may reflect the high care coordination and communication needs inherent to the gynecologic oncology population, particularly within a safety-net setting, where patients often require ongoing postoperative guidance despite proactive outreach. Standardized engagement with patients can lead to reassurance, improved satisfaction, and increased patient safety.

Although mortality did not differ statistically between groups, the numerically lower number of deaths in the phone-call cohort is notable. There were 3 deaths in the phone call group (1.4%), compared to 11 among those who were not called (4.4%) (*p* = 0.059). This trend, while not definitive, raises the possibility that earlier identification of complications, enhanced patient engagement, or improved care coordination may contribute to better outcomes. These findings warrant further exploration in a larger sample as our study was not powered to see a difference.

Importantly, this intervention may improve health equity. Postoperative contact improves access to care and allows patients to remain engaged during a vulnerable period after discharge (Boitano et al., 2023). In surgical populations, barriers such as limited health literacy, transportation challenges, and difficulty navigating the healthcare system can contribute to worse outcomes (Boitano et al., 2023; Theiss et al., 2022). For example, a study of colorectal surgery patients found that patients with limited health literacy had higher rates of complications, including surgical site infections (Theiss et al., 2022). By proactively reaching out to patients, answering questions, reinforcing discharge instructions, and providing tailored education, standardized phone calls may help bridge these gaps. This approach may be particularly valuable in safety-net settings, where patients are more likely to face socioeconomic barriers, by facilitating earlier recognition of complications and improving adherence to postoperative care plans (Boitano et al., 2023; Goehner et al., 2021).

There are several limitations to this study. Data were collected retrospectively to evaluate the efficacy of the intervention, introducing potential selection and documentation biases. Patients were categorized on whether they engaged in a phone call, rather than if they had their surgery in the pre- or post-intervention cohort, which may introduce some bias. Additionally, timing and delays in adjuvant therapy were not evaluated, and comparison of these outcomes would be challenging given the heterogeneity of diagnoses, histologies, and treatment indications within the study population. Finally, readmission rates were determined based on documentation available in our institution's medical record as well as other institutions participating in Care Everywhere (health information exchange feature within Epic electronic medical records that allows participating healthcare organizations to securely share patient records with one another), consequently, this likely did not capture all events as some readmissions occurring outside these networks were likely missed.

Overall, our findings support the value of standardized postoperative phone calls in improving follow up adherence and facilitating outpatient management of postoperative concerns. While the intervention did not reduce ED visits or readmissions outright, its association with fewer recurrent readmissions and improved outpatient management suggests that postoperative calls may play an important role in care coordination. Future studies should examine the cost-effectiveness of such interventions and explore whether tailoring phone call content to specific diagnoses or procedure types further enhances outcomes. Additionally, future studies should evaluate whether standardized postoperative outreach influences timing of adjuvant therapy initiation and treatment delays, particularly within specific gynecologic oncology disease groups.

## 5 Conclusion

Standardized postoperative phone calls represent a feasible, low cost intervention that improves follow up adherence and facilitates outpatient management of complications among gynecologic oncology patients. This approach may be particularly impactful in safety-net settings by improving access to care and supporting more equitable postoperative outcomes. Future prospective studies are warranted to further evaluate long term outcomes, cost-effectiveness, and opportunities to tailor interventions to specific patient populations and surgical procedures.

## CRediT authorship contribution statement

**Lilla Markel:** Writing – review & editing, Writing – original draft, Investigation, Data curation, Conceptualization. **Lauren Ellis:** Writing – original draft, Data curation. **Aubrey Wetzelberger:** Data curation, Conceptualization. **Matthew L. Anderson:** Writing – review & editing. **Thomas Rutherford:** Writing – review & editing. **Adrian Kohut:** Writing – review & editing. **Maya Yasukawa:** Writing – review & editing. **Emily Coughlin:** Formal analysis. **Rahul Mhaskar:** Formal analysis. **Vaagn Andikyan:** Writing – review & editing, Supervision, Conceptualization.

## Declaration of competing interest

The authors declare that they have no known competing financial interests or personal relationships that could have appeared to influence the work reported in this paper.

## Appendix A. Standardized questions

1. Are you experiencing any fevers or chills?

2. Are you experiencing pale/clammy skin, low blood pressure, fast heart rate, lightheadedness, fainting/loss of consciousness?

3. Any nausea or vomiting?

4. Would you describe your urination and bowel movements as normal or abnormal? Please, describe.

5. Are you passing gas?

6. How does the incision look?

7. Are you taking anything to manage your pain? (should be using Tylenol and Ibuprofen around the clock and only adding narcotic if pain is still not managed).

8. Are you experiencing any vaginal discharge/bleeding?

9. Follow up appointment?
